# Association of Sodium-Glucose Cotransporter 2 Inhibitors (SGLT2i) with Cardiac Arrhythmias: A Systematic Review and Meta-Analysis of Cardiovascular Outcome Trials

**DOI:** 10.31083/j.rcm2409258

**Published:** 2023-09-18

**Authors:** Xujie Wang, Xuexue Zhang, Wantong Zhang, Jiaxi Li, Weiliang Weng, Qiuyan Li

**Affiliations:** ^1^Xiyuan Hospital, China Academy of Chinese Medical Sciences, 100091 Beijing, China; ^2^National Clinical Research Center for Chinese Medicine Cardiology, 100091 Beijing, China; ^3^Institute of Clinical Pharmacology, China Academy of Chinese Medical Sciences, 100091 Beijing, China; ^4^The First Clinical College, Shanxi University of Chinese Medicine, 030024 Taiyuan, Shanxi, China

**Keywords:** sodium-glucose cotransporter 2 inhibitors, arrhythmia, tachycardia, tachyarrhythmia, bradycardia, bradyarrhythmia, cardiac arrest, meta-analysis, systematic review

## Abstract

**Background:**

Sodium-glucose cotransporter 2 inhibitors (SGLT2i) are a class of widely used hypoglycemic agents for the treatment of type 2 diabetes mellitus (T2DM). In addition to lowering blood glucose, SGLT2i protects the heart and kidney, significantly reduces cardiovascular events, and delays the progression of heart failure and chronic kidney disease. However, previous studies have not exhaustively discussed the association between SGLT2i and the risk of developing cardiac arrhythmias. The purpose of this study is to assess the association of SGLT2i with cardiac arrhythmias in patients with T2DM and without T2DM in cardiovascular outcome trials (CVOTs).

**Methods:**

We performed a meta-analysis and systematic review of CVOTs that compared SGLT2i with placebo. MEDLINE, Web of Science, The Cochrane Library and Embase were systematically searched from inception to December 2022. We included CVOTs reporting cardiovascular or renal outcomes with a follow-up duration of at least 6 months.

**Results:**

A total of 12 CVOTs with 77,470 participants were included in this meta-analysis (42,016 SGLT2i vs 35,454 control), including patients with T2DM, heart failure (HF), or chronic kidney disease (CKD). Follow-up duration ranged from 9 months to 5.65 years. Medications included empagliflozin, canagliflozin, dapagliflozin and ertugliflozin. SGLT2i were associated with a lower risk of tachycardia (risk ratio (RR) 0.86; 95% confidence interval (CI) 0.79–0.95), supraventricular tachycardia (SVT; RR 0.84; 95% CI 0.75–0.94), atrial fibrillation (AF; RR 0.86; 95% CI 0.75–0.97) and atrial flutter (AFL; RR 0.75; 95% CI 0.57–0.99) in patients with T2DM, HF and CKD. SGLT2i could also reduce the risk of cardiac arrest in CKD patients (RR 0.50; 95% CI 0.26–0.95). Besides, SGLT2i therapy was not associated with a lower risk of ventricular arrhythmia and bradycardia.

**Conclusions:**

SGLT2i therapy is associated with significantly reduced the risk of tachycardia, SVT, AF, and AFL in patients with T2DM, HF, and CKD. In addition, SGLT2i could also reduce the risk of cardiac arrest in CKD patients. Further researches are needed to fully elucidate the antiarrhythmic mechanism of SGLT2i.

## 1. Introduction

Sodium-glucose cotransporter 2 inhibitors (SGLT2i) are widely used to treat type 2 diabetes mellitus (T2DM) by inhibiting sodium and glucose reabsorption in the renal proximal convoluted tubules, resulting in natriuresis and glucosuria, promoting osmotic diuresis and lowering blood glucose [1]. SGLT2i were originally developed as novel hypoglycemic agents, but, with the deepening of research, these drugs have been discovered to have a wide range of additional effects, such as lowering body weight, metabolic shift, and reducing inflammatory responses [2].

In order to determine the association between new antidiabetic therapies and cardiovascular risk, the US Food and Drug Administration has set criteria for cardiovascular safety trials, which were also known as cardiovascular outcome trials (CVOTs) [3]. Recently, the advent of many large COVTs for SGLT2i has provided more information to optimize the treatment of T2DM, heart failure (HF) and chronic kidney disease (CKD), and to potentially reduce the risk of cardio-renal complications [4]. Compared with conventional randomized controlled trials (RCTs), COVTs are more helpful to identify the potential cardiovascular risk of drugs and their cardiovascular benefits. CVOTs are mostly RCTs with follow-up of more than 2 years. They included patients with confirmed cardiovascular disease (CVD) or at high risk of CVD. The primary endpoint of COVTs is major adverse cardiovascular events (MACE), and the degree of cardiovascular risk and follow-up duration of patients are important influencing factors.

Diabetes mellitus is a known risk factor for various cardiac arrhythmias, such as supraventricular tachycardia (SVT), ventricular arrhythmias (VA), and cardiac arrest, while bradyarrhythmias are also common in diabetic patients [5, 6, 7, 8]. Pathophysiological remodeling of cardiac function occurs at multiple levels in patients with HF, and abnormal changes in ion channels are likely to lead to various cardiac arrhythmias [9, 10]. CKD can also lead to increased cardiovascular risk such as cardiac arrhythmias and HF [11]. The present study identified potential antiarrhythmic benefits of SGLT2i, which can reduce cardiac arrhythmias by affecting cellular calcium ion current, calcium homeostasis, and reducing oxidative stress [12]. Therefore, the aim of this meta-analysis and systematic review was to gather the study results from all large CVOTs with SGLT2i to evaluate the effect of SGLT2i on common cardiac arrhythmia outcomes (tachycardia, SVT, VA, cardiac arrest, and bradyarrhythmia) in patients with T2DM, HF, and CKD.

## 2. Methods

This systematic review and meta-analysis were designed and conducted in accordance with the Cochrane Handbook of Systematic Reviews for interventions [13] and the Preferred Reporting Items for Systematic Reviews and Meta-Analyses (PRISMA) 2020 statement [14]. The PRISMA checklist is shown in **Supplementary Table 1**. This study was registered in the International Prospective Register of Systematic Reviews (PROSPERO), with the ID number CRD42023405574.

### 2.1 Data Sources and Search Strategy

To identify all COVTs, we systematically explored MEDLINE (PubMed), Web of Science, The Cochrane Library, and Embase from database inception to December 10, 2022. Data from included clinical trials was obtained from ClinicalTrials.gov (https://clinicaltrials.gov/). In this study, medical subject headings (MeSH) strategy was used to search the literature using a combination of MeSH major topics and entry terms. The search terms were as follows: sodium-glucose transporter 2 inhibitors, SGLT2 inhibitor, SGLT2 inhibitors, SGLT 2 inhibitors, SGLT-2 inhibitors, sodium-glucose transporter 2 inhibitor, sodium glucose transporter 2 inhibitor, sodium glucose transporter 2 inhibitors, gliflozins, gliflozin, SGLT-2 inhibitor, SGLT 2 inhibitor, empagliflozin, dapagliflozin, canagliflozin, sotagliflozin, ertugliflozin, henagliflozin, randomized controlled trial, randomized, placebo, type 2 diabetes mellitus, chronic kidney disease and heart failure. Detailed search strategies are presented in **Supplementary Table 2**.

### 2.2 Inclusion and Exclusion Criteria

There was no restriction with respect to the language, date of publication, or publication status. To determine the antiarrhythmic effect of SGLT2i, we excluded trials where patients received combination therapy, and selected placebo as the comparator. We included RCTs comparing SGLT2i with placebo in adult patients with T2DM, HF or CKD and reported outcomes of interest as cardiac arrhythmias. Studies that met the inclusion criteria were included: (1) RCTs comparing any SGLT2i with placebo; (2) RCTs met FDA guidance for cardiovascular safety trials; (3) follow-up duration of at least six months; (4) participants aged 18 years or older with diagnosed T2DM and/or HF and/or CKD. Exclusion criteria: (1) studies without cardiac arrhythmia outcomes and those with duplicate data; (2) reviews, case reports, conference abstracts, and other non-RCT studies; (3) studies where data are incomplete or cannot be converted into usable data.

### 2.3 Outcomes of Interest

In this study, outcomes of interest were reported as a serious adverse event according to Medical Dictionary for Regulatory Activities (MedDRA version 18.0) to reduce potential bias. Primary outcomes were incidence of tachycardia, supraventricular tachycardia, ventricular arrhythmia, cardiac arrest and bradyarrhythmia. Tachycardia in this meta-analysis included SVT and VA. SVT in this meta-analysis included sinus arrhythmia/tachycardia, atrial tachycardia, atrial fibrillation and atrial flutter. VA in this meta-analysis included ventricular tachycardia, torsade de pointes, ventricular extrasystoles, ventricular flutter and ventricular fibrillation. Bradyarrhythmia in this meta-analysis included sinus node dysfunction (SND), atrioventricular block (AVB) and conduction tissue disease (CTD).

### 2.4 Data Extraction and Quality Assessment

Data search and extraction were performed independently by 2 investigators (XJW and XXZ). Any disagreements were resolved by author consensus or by consulting last author (QYL). The data extraction content included: study title, year of publication, ClinicalTrials.gov identifier, research type, study population, sample size, patients characteristics (age and follow-up duration), treatment information (type of SGLT2i and dose) and outcome data (number of events for each cardiac arrhythmia outcome).

According to the recommendations of the Cochrane Collaboration, Revised Cochrane risk-of-bias tool for randomized trials (ROB 2) [15] was used to assess the risk of bias of included studies. Risk of bias was assessed across five distinct domains, including: bias arising from the randomization process, bias due to deviations from intended interventions, bias due to missing outcome data, bias in measurement of the outcome and bias in selection of the reported result. Judgments within the five domains mentioned above will lead to an overall risk of bias judgment, and these answers lead to judgments of low risk of bias, some concern, or high risk of bias.

### 2.5 Data Synthesis and Analysis

Statistical analysis was performed using Cochrane ReviewManager (RevMan) 5.3 (The Cochrane Collaboration, Copenhagen, Denmark). The extracted data were dichotomized data, so the risk ratio (RR) of the data was evaluated with a 95% confidence interval (CI). Heterogeneity across studies was tested by using Q test and $I^{2}$ statistic. According to the heterogeneity among various studies, the Mantel-Haenszel equation and the fixed or random effect model were used to analyze the combined data.

$I^{2}$ index values less than 50% were considered low heterogeneity, 51% to 75% were considered moderate heterogeneity, and greater than 75% were considered high heterogeneity. In addition, publication bias was assessed by visual inspection of the funnel plot.

## 3. Results

### 3.1 Characteristics of Eligible Studies

**Fig. 1.**
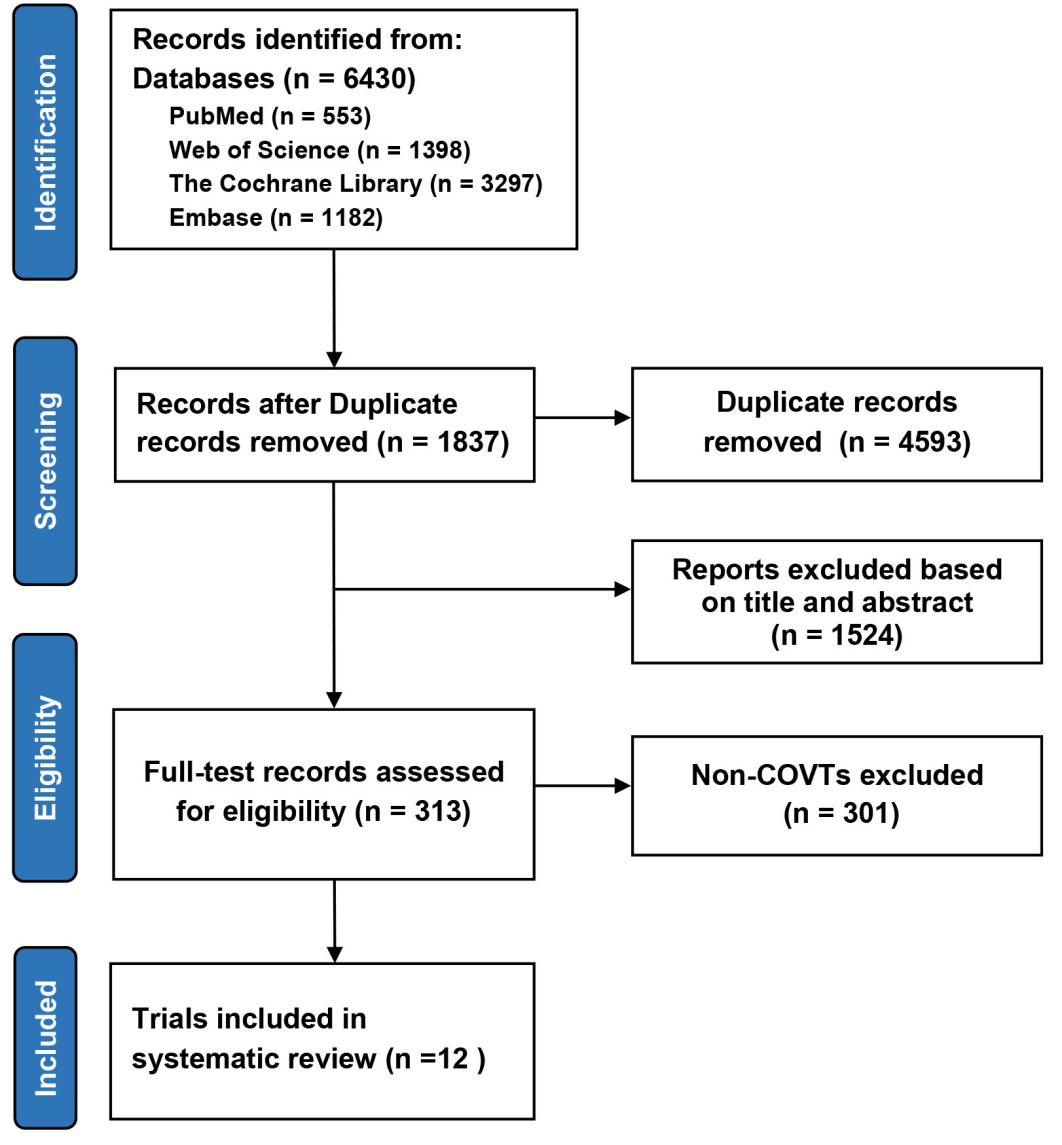
**Preferred reporting items for meta-analysis and systematic review flowchart of selection**. CVOTs, cardiovascular outcome trials.

Among the 1721 citations identified by literature search, 12 COVTs from 11 articles [16, 17, 18, 19, 20, 21, 22, 23, 24, 25, 26] were included in this study (Fig. 1). All trials were multinational, rigorously parallel-group, double-blind designed RCTs and sponsored by the industry. Publication of above studies ranged from 2015 to 2021, with 4 studies published in 2020. A total of 77,470 participants were included in this meta-analysis, including patients with T2DM, HF, or CKD. Follow-up duration ranged from 9 months to 5.65 years. The baseline characteristics of the trials included were summarized in Table 1.

**Table 1.** **Characteristics of the included COVTs**.

Study (Clinical Trial)	Year	Study design (Study IDs)	Study population	Type of SGLT2i (dose, mg)	No. of patients		Age, mean (SD) or median, y	Follow-up, y	Primary outcome
					SGLT2i group	Placebo group			
EMPA-REG OUTCOME	2015	RCT (NCT01131676)	T2DM with high CV risk	Empagliflozin (10 and 25)	4687	2333	63.1 (8.7)	2.95 (mean)	MACE
CANVAS	2017	RCT (NCT01032629)	T2DM with history or high risk of CVE	Canagliflozin (100 and 300)	2888	1442	62.4 (8.02)	5.65 (mean)	MACE
CANVAS-R	2017	RCT (NCT01989754)	T2DM with history or high risk of CVE	Canagliflozin ${\mathrm{(100 and 300)}}^{※}$	2907	2905	64 (8.35)	2.07 (meam)	MACE
DECLARE-TIMI 58	2018	RCT (NCT01730534)	T2DM with history or high risk of CVD	Dapagliflozin (10)	8582	8578	63.9 (6.8)	4.2 (median)	MACE
CREDENCE	2019	RCT (NCT02065791)	T2DM and ACKD	Canagliflozin (100)	2202	2199	63 (9.2)	2.62 (median)	ESKD, doubling of serum creatinine level, Or death from renal or CV causes
DAPA-HF	2019	RCT (NCT03036124)	HFrEF with or without T2DM	Dapagliflozin (5 or 10)	2373	2371	66.3 (10.9)	1.52 (median)	worsening HF or CV death
DAPA-CKD	2020	RCT (NCT03036150)	CKD with or without T2DM	Dapagliflozin (10)	2152	2152	61.8 (12.1)	2.4 (median)	eGFR decline ≥50%, ESKD, or death from renal or CV causes
VERTIS CV	2020	RCT (NCT01986881)	T2DM and CVD	Ertugliflozin (5 or 15)	5499	2747	64.4 (8.1)	3.5 (mean)	MACE
EMPEROR-Reduced	2020	RCT (NCT03057977)	HFrEF with or without T2DM	Empagliflozin (10)	1863	1867	66.8 (11)	1.33 (median)	CV death or hospitalization for HF
SCORED	2020	RCT (NCT03315143)	T2DM, CKD and risk for CVD	Sotagliflozin (200 or 400)	5292	5292	69	1.33 (median)	MACE, CV death and hospitalization for HF
SOLOIST-WHF	2021	RCT (NCT03521934)	T2DM and HF	Sotagliflozin (200 or 400)	608	614	70	0.75 (median)	CV death, urgent visit or hospitalization for HF
EMPEROR-Preserved	2021	RCT (NCT03057951)	HFpEF with or without T2DM	Empagliflozin (10)	2997	2991	71.9 (9.4)	2.18 (median)	CV death and hospitalization for HF

CVOTs, cardiovascular outcome trials; T2DM, type 2 diabetes mellitus; CV, cardiovascular; CVD, cardiovascular disease; CVE, cardiovascular events; MACE, major adverse cardiovascular events; HF, heart failure; HFpEF, heart failure with preserved ejection fraction; HFrEF, heart failure with reduced ejection fraction; ACKD, albuminuric chronic kidney disease; ESKD, end-stage kidney disease; eGFR, estimated glomerular filtration rate; CKD, chronic kidney disease; SGLT2i, sodium-glucose cotransporter 2 inhibitors. ※: 100 mg for first 13 weeks then 300 mg.

After quality assessment of all studies according to ROB 2’s evaluation process, we judged all 12 COVTs were low risk of bias (**Supplementary Fig. 1**). Due to the low heterogeneity across various studies, fixed effect model was used for all comparisons.

### 3.2 Tachycardia

**Fig. 2.**
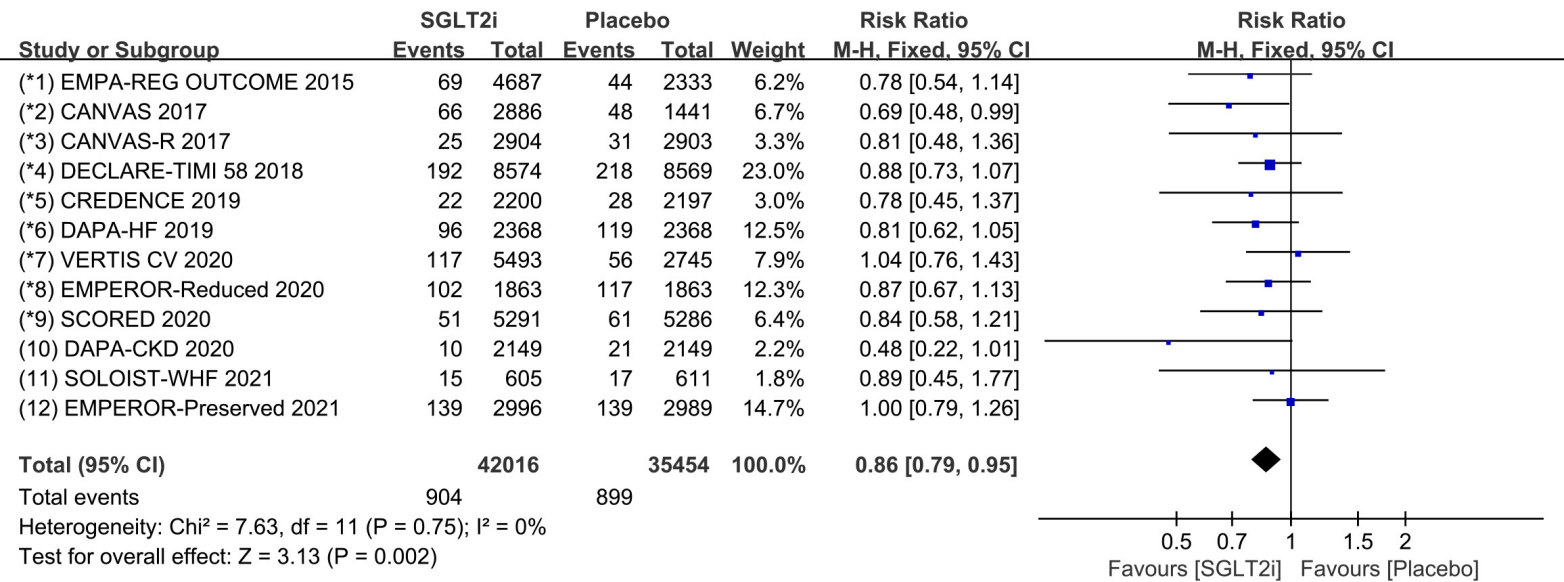
**The pooled effect of tachycardia incidence**. SGLT2i, sodium-glucose cotransporter 2 inhibitors.

Tachycardia is a general term for tachyarrhythmia, including supraventricular tachycardia and ventricular arrhythmia. All 12 COVTs reported the incidence of tachycardia. The risk of tachycardia in the SGLT2i group was significantly lower than that in the placebo group (Fig. 2; RR, 0.86; 95% CI: 0.79 to 0.95; *p* = 0.002; $I^{2}$ = 0%). Treatment with SGLT2i was associated with a 14% reduction in the incidence of tachycardia compared to the placebo group. Publication bias for tachycardia was presented in funnel plot (**Supplementary Fig. 2**).

#### 3.2.1 Supraventricular Tachycardia

**Fig. 3.**
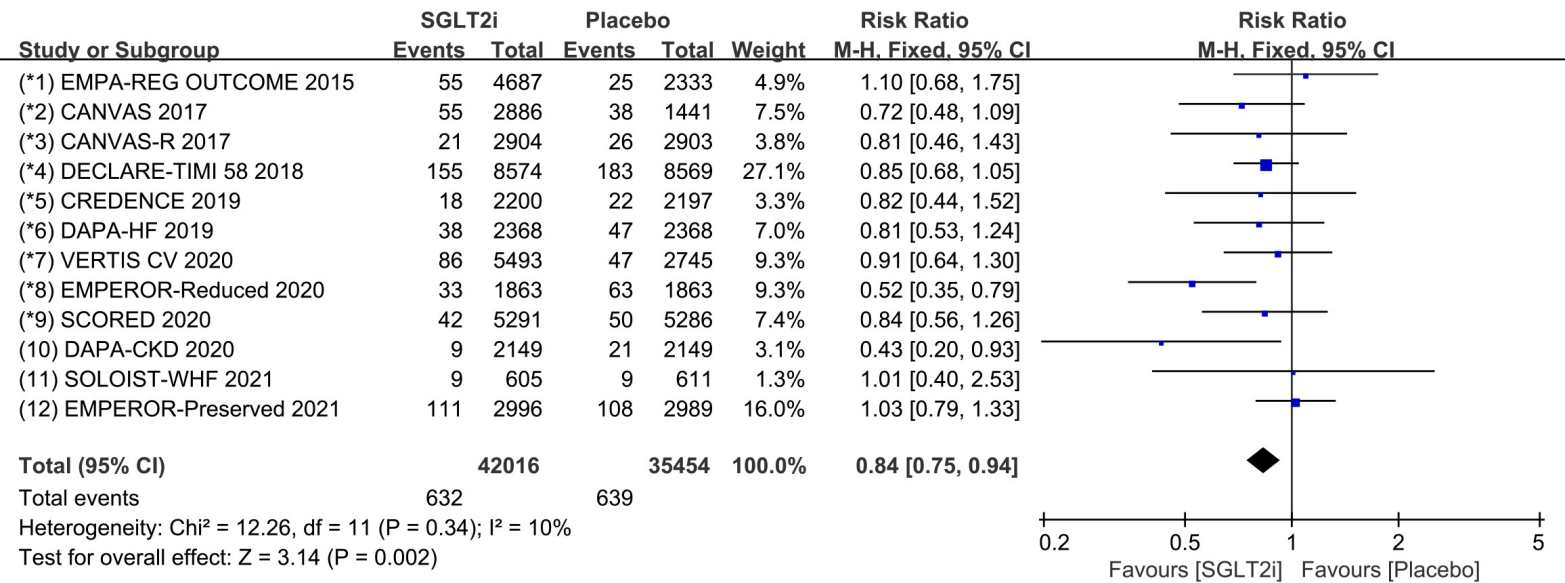
**SVT events with SGLT2i vs placebo in patients with T2DM, HF or CKD**. SGLT2i, sodium-glucose cotransporter 2 inhibitors; SVT, supraventricular tachycardia; T2DM, type 2 diabetes mellitus; HF, heart failure; CKD, chronic kidney disease.

Raw data on supraventricular tachycardia, sinus arrhythmia, sinus tachycardia, atrial tachycardia, atrial fibrillation and atrial flutter from clinical trials were included in the meta-analysis of supraventricular tachycardia. All 12 COVTs reported the incidence of supraventricular tachycardia. The risk of supraventricular tachycardia in the SGLT2i group was significantly lower than that in the placebo group (Fig. 3; RR, 0.84; 95% CI: 0.75 to 0.94; *p* = 0.002; $I^{2}$ = 10%). Treatment with SGLT2i was associated with a 16% reduction in the incidence of supraventricular tachycardia compared to the placebo group. Publication bias for supraventricular tachycardia was presented in funnel plot (**Supplementary Fig. 3**).

**Fig. 4.**
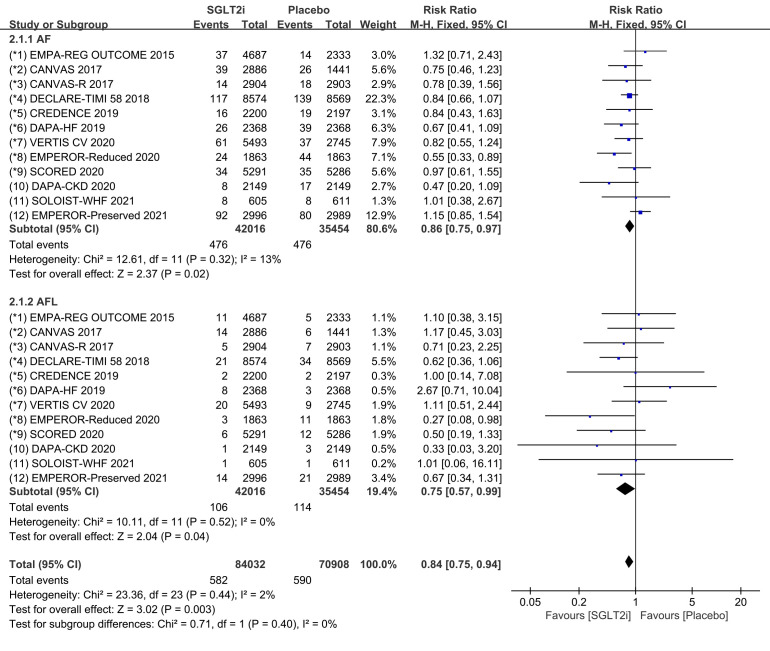
**AF and AFL events with SGLT2i vs placebo in patients with T2DM, HF or CKD**. AF, atrial fibrillation; AFL, atrial flutter; SGLT2i, sodium-glucose cotransporter 2 inhibitors; T2DM, type 2 diabetes mellitus; HF, heart failure; CKD, chronic kidney disease.

In addition, we performed subgroup analyses for the incidence of atrial fibrillation and atrial flutter. All 12 COVTs reported the incidence of atrial fibrillation and atrial flutter. The risk of atrial fibrillation in the SGLT2i group was significantly lower than that in the placebo group (Fig. 4; RR, 0.86; 95% CI: 0.75 to 0.97; *p* = 0.02; $I^{2}$ = 13%). The risk of atrial flutter in the SGLT2i group was significantly lower than that in the placebo group (Fig. 4; RR, 0.75; 95% CI: 0.57 to 0.99; *p* = 0.04; $I^{2}$ = 0%). SGLT2i therapy reduced the incidence of atrial fibrillation and flutter by 14% and 25%, respectively, compared with placebo. Publication bias for atrial fibrillation (AF) and atrial flutter (AFL) was presented in funnel plot (**Supplementary Fig. 4**).

#### 3.2.2 Ventricular Arrhythmia

**Fig. 5.**
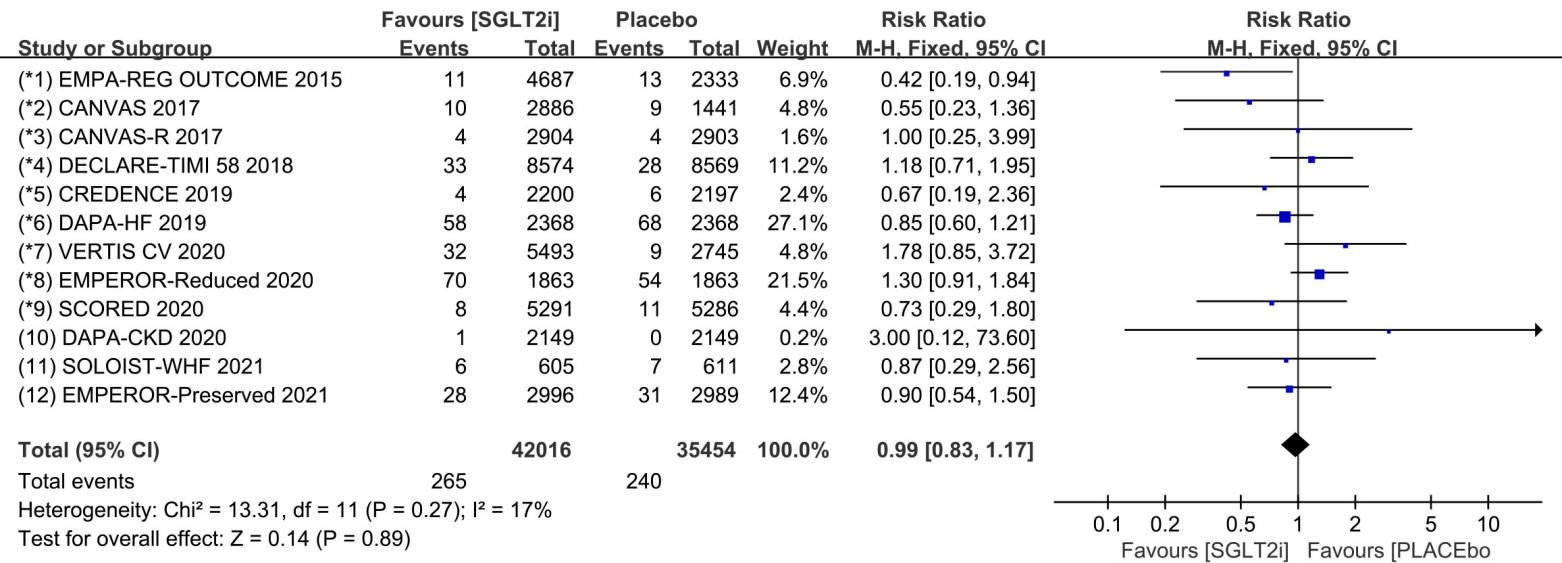
**VA events with SGLT2i vs placebo in patients with T2DM, HF or CKD**. VA, ventricular arrhythmias; SGLT2i, sodium-glucose cotransporter 2 inhibitors; T2DM, type 2 diabetes mellitus; HF, heart failure; CKD, chronic kidney disease.

Raw data on ventricular arrhythmia, ventricular tachycardia, torsade de pointes, ventricular extrasystoles, ventricular flutter and ventricular fibrillation from clinical trials were included in the meta-analysis of ventricular arrhythmia. All 12 COVTs reported the incidence of ventricular arrhythmia. The risk of ventricular arrhythmia in the SGLT2i group was not significantly different from that in the placebo group (Fig. 5; RR, 0.98; 95% CI: 0.82 to 1.16; *p* = 0.79; $I^{2}$ = 13%). Publication bias for ventricular arrhythmia was presented in funnel plot (**Supplementary Fig. 5**).

**Fig. 6.**
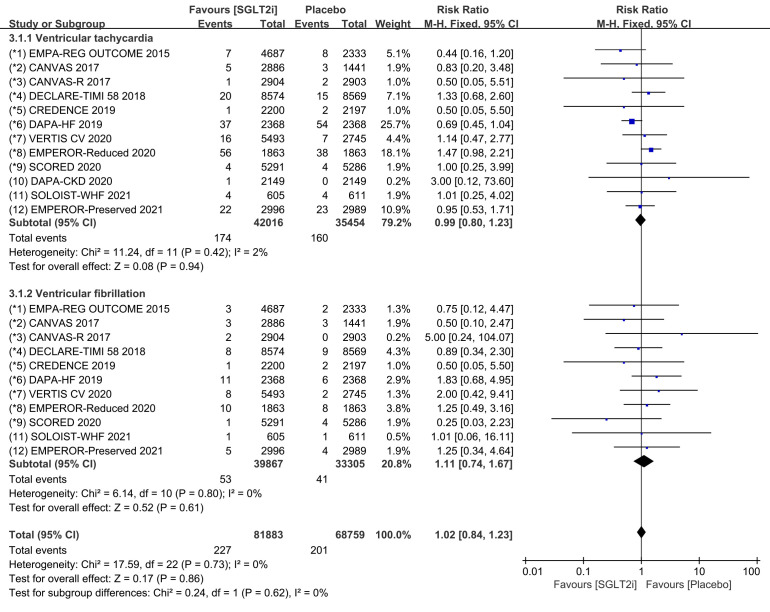
**Ventricular tachycardia and ventricular fibrillation events with SGLT2i vs placebo in patients with T2DM, HF or CKD**. SGLT2i, sodium-glucose cotransporter 2 inhibitors; T2DM, type 2 diabetes mellitus; HF, heart failure; CKD, chronic kidney disease.

Subgroup analyses assessed the incidence of ventricular tachycardia (including ventricular tachyarrhythmia and torsade de pointes) and ventricular fibrillation, respectively. The incidence of ventricular tachycardia was reported in all 12 COVTs, and the incidence of ventricular fibrillation was reported in 11 COVTs. However, the risk of ventricular tachycardia (Fig. 6; RR, 0.97; 95% CI: 0.78 to 1.20; *p* = 0.78; $I^{2}$ = 3%) and ventricular fibrillation (Fig. 6; RR, 1.11; 95% CI: 0.74 to 1.67; *p* = 0.61; $I^{2}$ = 0%) in the SGLT2i group was not significantly different from that in the placebo group. Publication bias for ventricular tachycardia and ventricular fibrillation was presented in funnel plot (**Supplementary Fig. 6**).

### 3.3 Cardiac Arrest

**Fig. 7.**
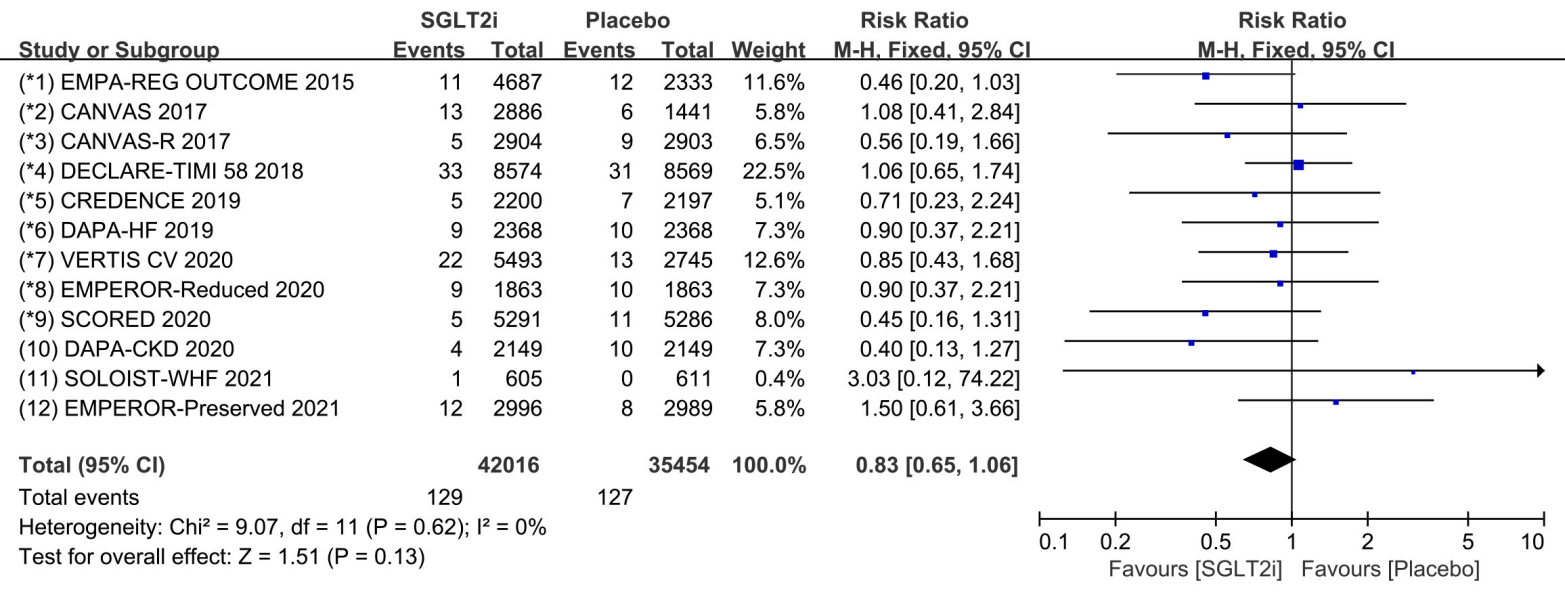
**The pooled effect of cardiac arrest incidence**. SGLT2i, sodium-glucose cotransporter 2 inhibitors.

All 12 COVTs reported the incidence of cardiac arrest. The risk of cardiac arrest in the SGLT2i group was not significantly different from that in the placebo group (Fig. 7; RR, 0.83; 95% CI: 0.65 to 1.06; *p* = 0.13; $I^{2}$ = 0%). Publication bias for cardiac arrest was presented in funnel plot (**Supplementary Fig. 7**).

**Fig. 8.**
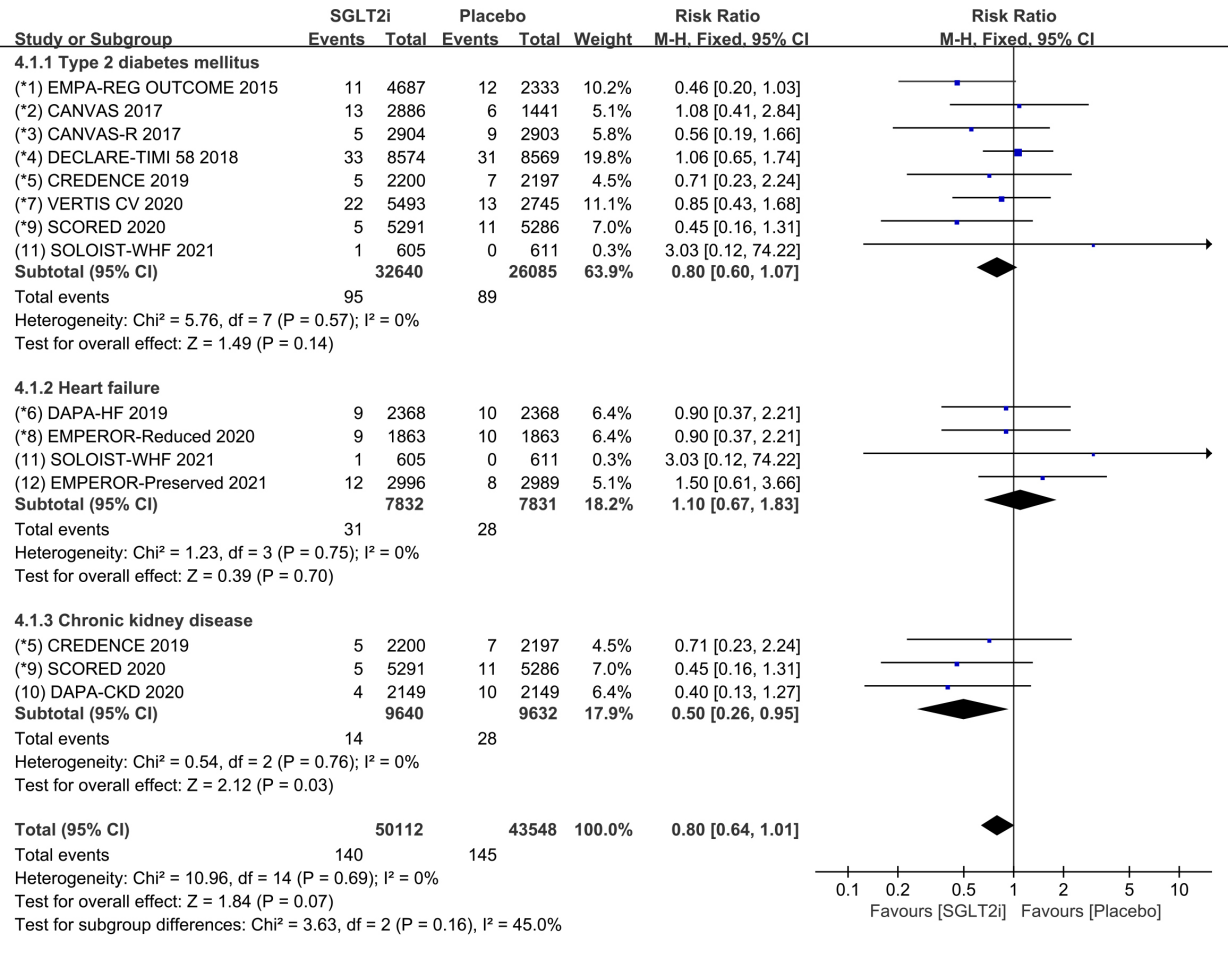
**Cardiac arrest events with SGLT2i vs placebo in patients with T2DM, HF or CKD**. SGLT2i, sodium-glucose cotransporter 2 inhibitors; T2DM, type 2 diabetes mellitus; HF, heart failure; CKD, chronic kidney disease.

Subgroup analyses were conducted to evaluate the prevalence of cardiac arrest in patients with T2DM, HF, and CKD. 8 COVTs reported the incidence of cardiac arrest in T2DM patients. The results showed that the risk of cardiac arrest in the SGLT2i group was not significantly different from that in the placebo group (Fig. 8; RR, 0.80; 95% CI: 0.60 to 1.07; *p* = 0.14; $I^{2}$ = 0%). The incidence of cardiac arrest in HF patients was reported in 4 COVTs, the risk of cardiac arrest in the SGLT2i group was not significantly different from that in the placebo group (Fig. 8; RR, 1.10; 95% CI: 0.67 to 1.83; *p* = 0.70; $I^{2}$ = 0%). For CKD patients, the incidence of cardiac arrest was reported in 3 COVTs, and the risk of cardiac arrest in the SGLT2i group was significantly lower than that in the placebo group (Fig. 8; RR, 0.50; 95% CI: 0.26 to 0.95; *p* = 0.03; $I^{2}$ = 0%). Compared with placebo, the use of SGLT2i therapy reduced the incidence of cardiac arrest in CKD patients by 50%. Publication bias for cardiac arrest in patients with T2DM, HF and CKD was assessed separately and shown in funnel plot (**Supplementary Fig. 8**).

### 3.4 Bradycardia

**Fig. 9.**
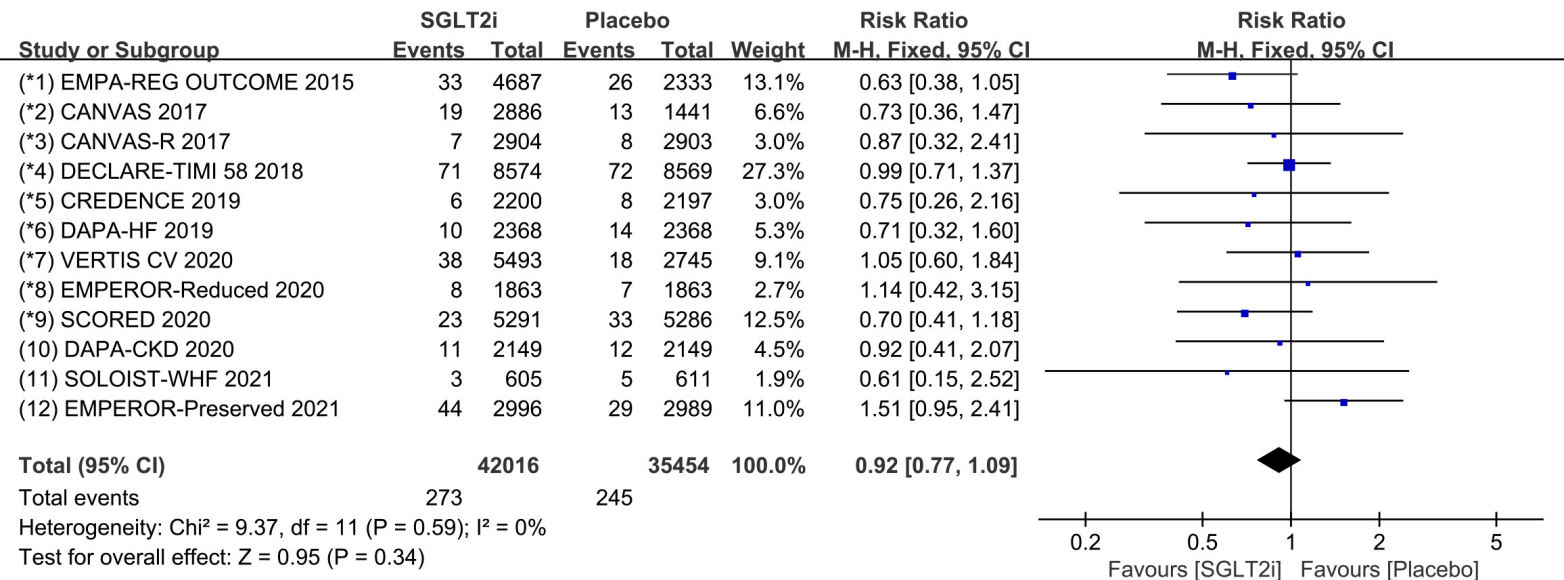
**The pooled effect of bradycardia incidence**. SGLT2i, sodium-glucose cotransporter 2 inhibitors.

Raw data on SND, AVB and CTD from clinical trials were included in the meta-analysis of bradycardia. All 12 COVTs reported the incidence of bradycardia. The risk of bradycardia in the SGLT2i group was not significantly different from that in the placebo group (Fig. 9; RR, 0.92; 95% CI: 0.77 to 1.09; *p* = 0.34; $I^{2}$ = 0%). Publication bias for bradycardia was presented in funnel plot (**Supplementary Fig. 9**).

**Fig. 10.**
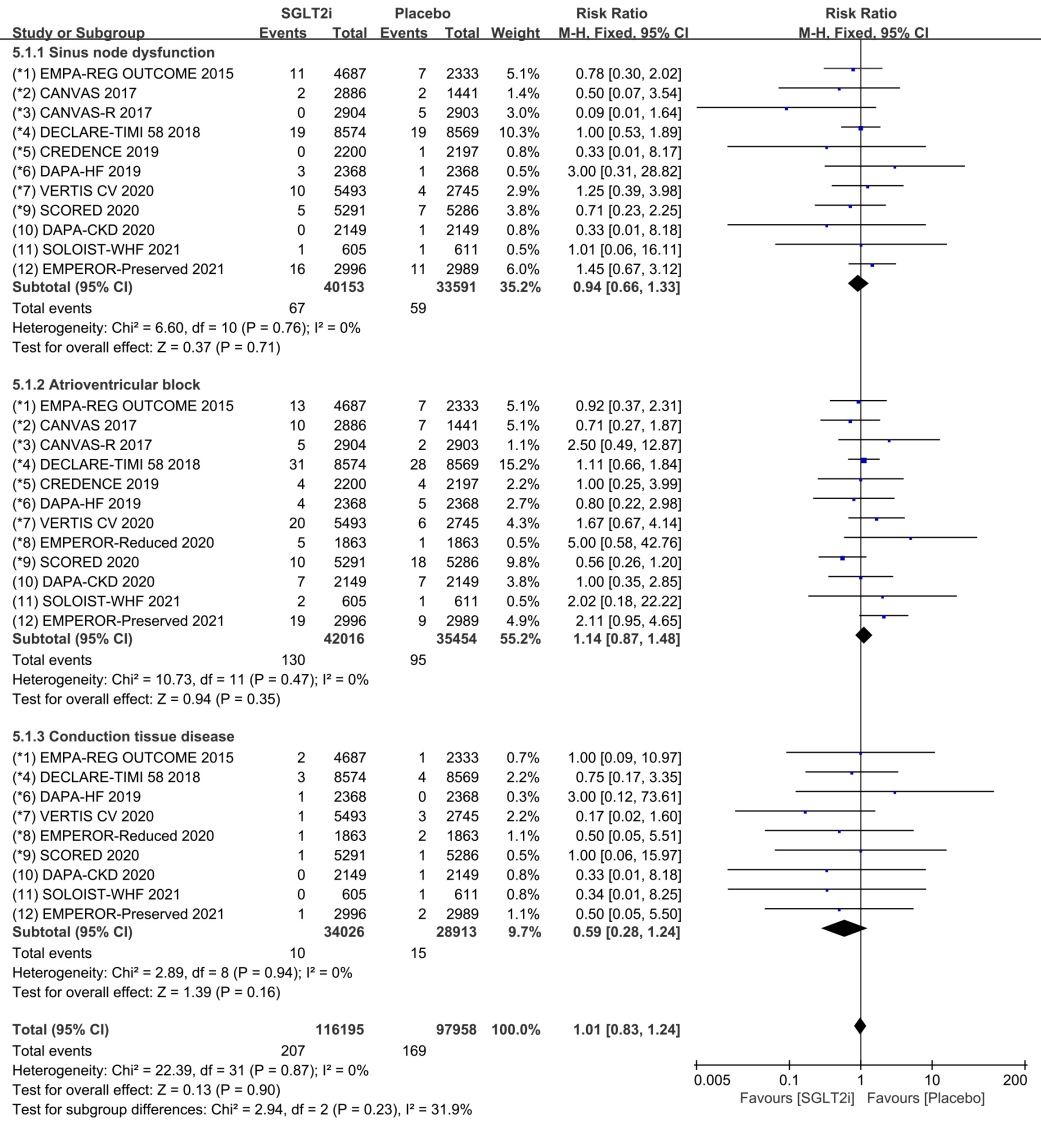
**SND, AVB and CTD events with SGLT2i vs placebo in patients with T2DM, HF or CKD**. SND, sinus node dysfunction; AVB, atrioventricular block; CTD, conduction tissue disease; SGLT2i, sodium-glucose cotransporter 2 inhibitors; T2DM, type 2 diabetes mellitus; HF, heart failure; CKD, chronic kidney disease.

Subgroup analyses were conducted to assess the incidence of SND, AVB and CTD, respectively. Raw data on SND, sinus bradycardia and sinus arrest from clinical trials were included in the meta-analysis of SND. The incidence of SND was reported in 11 COVTs, and the risk of SND in the SGLT2i group was not significantly different from that in the placebo group (Fig. 10; RR, 0.94; 95% CI: 0.66 to 1.33; *p* = 0.71; $I^{2}$ = 0%).

Raw data on AVB, AVB first degree, AVB second degree and AVB complete from clinical trials were included in the meta-analysis of AVB. The incidence of AVB was reported in 12 COVTs, and the risk of AVB in the SGLT2i group was not significantly different from that in the placebo group (Fig. 10; RR, 1.14; 95% CI: 0.87 to 1.48; *p* = 0.35; $I^{2}$ = 0%).

Raw data on bundle branch block right, bundle branch block left, bundle branch block bilatera, bifascicular block and trifascicular block from clinical trials were included in the meta-analysis of CTD. The incidence of CTD was reported in 9 COVTs, and the risk of CTD in the SGLT2i group was not significantly different from that in the placebo group (Fig. 10; RR, 0.59; 95% CI: 0.28 to 1.24; *p* = 0.16; $I^{2}$ = 0%). Publication bias for SND, AVB and CTD was presented in funnel plot (**Supplementary Fig. 10**).

## 4. Discussion

In this meta-analysis and systematic review of 12 COVTs involving 77,470 patients with T2DM, HF, or CKD at risk of developing cardiac arrhythmias, we found that SGLT2i therapy was associated with a significant reduction in the risk of tachycardia, SVT, AF and AFL in patients with T2DM, HF and CKD. Besides, SGLT2i therapy could also reduce the risk of cardiac arrest in CKD patients. Therefore, SGLT2i may have important therapeutic effects against above types of cardiac arrhythmias, and can effectively prevent these serious cardiovascular adverse events in patients with T2DM, HF, and CKD. However, there was no significant difference in the risks of VA (ventricular tachycardia and fibrillation), bradycardia (SND, AVB, and CTD), and cardiac arrest in patients with T2DM and HF in the SGLT2i group compared to the placebo group. Nevertheless, we cannot rule out the potential therapeutic effect of SGLT2i on these cardiac arrhythmias, and more COVTs need to be included in the future to validate these findings.

SGLT2 inhibitors have a direct effect on cardiomyocyte metabolism and can improve cardiac function by reducing JunD expression [27, 28]. SGLT2i can balance autonomic system activity, induce an ameliorative regulation of sympathetic systemic tone, and reduce the recurrence of vaso-vagal syncope in T2DM patients [29]. In addition, SGLT2i also has anti-inflammatory and antiarrhythmic properties in patients with acute coronary syndrome, stable ischemic heart disease, multi-vessel coronary stenosis, and can significantly reduce in-hospital arrhythmic burden in treated patients [30, 31, 32, 33]. For cardiac arrhythmias, SGLT2i have multiple antiarrhythmic mechanisms, which include: (1) osmotic diuresis to lower blood glucose and reduce cardiac load: both the osmotic effect of glucose and natriuresis contribute to the diuretic effect of SGLT2i, resulting in plasma volume contraction, which hemodynamically unload the left ventricle, decreases myocardial oxygen consumption, filling pressure, and ventricular wall tension [34]; (2) regulation of cardiac ion balance: SGLT2i can affect a variety of cardiac ion currents to attenuate action potential duration prolongation and reduce the development of calcium-related cardiac arrhythmias by affecting calcium homeostasis and calcium ion current [12]; (3) regulation of mitochondrial function and improvement of myocardial remodeling: SGLT2i can increases mitochondrial calcium uptake and mitigate mitochondrial swelling in cardiomyocytes, thereby restoring the antioxidant capacity of mitochondria and exerting antiarrhythmic effects [35]; and (4) inhibition of sympathetic activity: SGLT2i may reduce the risk of arrhythmias by inhibiting levels of markers of the sympathetic nervous system (norepinephrine and tyrosine hydroxylase), attenuating the stimulation of afferent sympathetic activity, and reducing the activity of sympathetic nervous system [36]. Additionally, SGLT2i can reduce cardiac inflammation, myocardial oxygen consumption, and oxidative stress, all of which may contribute to reducing the risk of cardiac arrhythmias [12].

Sinus tachycardia, atrial tachycardia, atrioventricular junctional tachycardia, atrioventricular re-entrant tachycardia, AF and AFL are common SVT in clinical practice [37]. The pathogenesis of SVT is due to abnormalities or enhanced automaticity of non-pacemaker cells and may also involve oscillations in membrane potential because of abnormal pulse initiation [38]. A meta-analysis of 32 RCTs found that SGLT2i significantly reduced the risk of atrial arrhythmias, which partially supports the findings of this study [39]. However, since this study only included results from patients with AF and AFL, its rigor is limited compared to other meta-analysis. As a more severe SVT, AF is listed as a separate clinical guideline by the European Society of Cardiology (ESC) and the American heart association (AHA) [40, 41]. The results of several studies also support the findings of this meta-analysis that SGLT2i significantly reduces the risk of AF and AFL [42, 43, 44].

Compared to SVT, VA belongs to a more serious arrhythmia, and severe VA can be life-threatening. Ventricular tachycardia, ventricular extrasystoles, ventricular flutter, and ventricular fibrillation are common VAs [45]. Abnormal automaticity or enhanced automaticity of subordinate pacemaker cells originating in the His-Purkinje system or ventricular myocardium can cause the development of VA. Changes in transporter function and/or expression or ion channel and intercellular coupling secondary to underlying pathology, are also mechanisms leading to changes in myocardial action potential [46]. Similar to the results of this meta-analysis, several previous studies have found that SGLT2i treatment was not associated with a reduced risk of VA [39, 42, 47]. However, one study [41] found that SGLT2i reduced the risk of ventricular tachycardia, so more CVOTs should be included in the future to verify the association between SGLT2i and the risk of VA.

Cardiac arrest is a severe clinical emergency that is defined by the ESC as cessation of normal cardiac activity with hemodynamic collapse [45]. Despite few studies on cardiac arrest, the results of a meta-analysis [44] have been consistent with the findings of this study, and no association has been found between SGLT2i and the risk of developing cardiac arrest. Interestingly, our subgroup analysis of different disease populations showed that SGLT2i therapy significantly reduced the risk of cardiac arrest in CKD patients. CKD patients often experience adverse cardiomyopathic and vasculopathic conditions, resulting in left ventricular pressure and volume overload, which increases the risk of cardiac arrest in CKD patients [48]. Therefore, the findings of this study could provide new research clues for preventing cardiac arrest in patients with CKD.

In contrast to tachycardia, bradycardia is a type of arrhythmia that causes heart rate to slow down. Degenerative fibrosis of the sinoatrial node, atria, atrioventricular node, or other conduction tissues can lead to bradyarrhythmias [49]. Although drug therapy for bradyarrhythmia is mostly used for acute management, complementary therapies like traditional Chinese medicine, provide a new treatment method for bradyarrhythmias [50, 51, 52], so it is necessary to continuously expand the drug therapy for bradyarrhythmias. Unfortunately, this meta-analysis found that SGLT2i therapy did not reduce the risk of bradycardia, such as sinoatrial dysfunction, atrioventricular block, and conduction tissue disease in patients with T2DM, HF and CKD. While a meta-analysis has reported that SGLT2i was associated with a lower risk of bradycardia [43]. But we browsed the full text and found that the results of this study were not rigorous, because they only included data with the term “Bradycardia” and numerous other original data were ignored. Therefore, more CVOTs should be conducted to verify the association between SGLT2i therapy and the risk of bradycardia in the future.

This meta-analysis has several limitations. First, none of the included COVTs described a systematic method to evaluate cardiac arrhythmias, and arrhythmic events were reported as serious adverse events rather than outcomes. Besides, the arrhythmia terms in COVTs were coded according to MedDRA, but the descriptions of each type of arrhythmia were mixed and not uniform, which may have biased the results of the study. Finally, the lack of standardized definitions for arrhythmia endpoints in individual studies may also contribute to reporting bias.

## 5. Conclusions

This meta-analysis and systematic review demonstrated that SGLT2i therapy is effective in reducing the risk of tachycardia, SVT, AF, and AFL in patients with T2DM, HF, and CKD. In addition, SGLT2i may also reduce the risk of cardiac arrest in patients with CKD. These findings provide robust evidence to support the use of SGLT2i in reducing the risk of cardiovascular disease in patients with T2DM, HF, and CKD. More prospective trials are needed to confirm the antiarrhythmic effect of SGLT2i and to further elucidate their underlying mechanisms.

## Supplementary Material

Supplementary material associated with this article can be found, in the online version, at https://doi.org/10.31083/j.rcm2409258.
